# Surface Treatment and Pre-Repair Thermal Aging Effects on Repair Bond Strength of Resin-Matrix CAD/CAM Ceramics

**DOI:** 10.3390/biomimetics11100696

**Published:** 2026-10-01

**Authors:** Şifa Oğuz, Akın Aladağ, Serdar Gözler

**Affiliations:** 1 Department of Prosthetic Dentistry, Faculty of Dentistry, Muğla Sıtkı Koçman University, 48000 Muğla, Türkiye; sifaoguz@mu.edu.tr (Ş.O.); akinaladag@mu.edu.tr (A.A.); 2 Department of Prosthodontics, Faculty of Dentistry, Istanbul Atlas University, 34000 Istanbul, Türkiye

**Keywords:** resin-matrix ceramic, CAD/CAM, repair, bond strength, surface treatment, thermocycling, tribochemical silica coating

## Abstract

Conservative repair motivates investigation of bonding to heterogeneous restorative substrates. This in vitro study evaluated initial composite repair shear bond strength (SBS) following surface conditioning of aged and non-aged resin-matrix CAD/CAM materials. VITA Enamic, GC Cerasmart, and Shofu Block HC received either 25,000 pre-repair thermal cycles or no thermocycling, followed by ground control, hydrofluoric acid (HF), Al_2_O_3_ abrasion, CoJet, or diamond bur treatment (*n* = 7/cell; 210 specimens). Following silane, universal adhesive, and composite application, SBS was tested at 1 mm/min. Three-way ANOVA identified surface treatment as the largest main effect (partial η^2^ = 0.694), with significant material × surface treatment (*p* < 0.001) and three-way (*p* = 0.016) interactions. The post-hoc pattern was particularly associated with Al_2_O_3_-treated GC Cerasmart: SBS decreased from 15.55 to 7.54 MPa after pre-repair aging (Bonferroni-adjusted *p* < 0.001); no statistically significant aging difference was detected in the other material–treatment combinations. No statistically significant HF–CoJet difference was detected within VITA Enamic or Shofu Block HC. Material-dependent pairwise patterns did not identify a single universally optimal surface-conditioning protocol across the tested materials. These findings concern initial repair bonding to previously aged substrates; post-repair durability and biomimetic tissue preservation were not evaluated.

## 1. Introduction

Resin-matrix CAD/CAM ceramics (RMCs) combine ceramic or inorganic filler phases with polymers, but their phase organization differs among products [1,2]. A relevant biological reference is the enamel–dentin complex, whose mechanically dissimilar constituents are integrated through the dentinoenamel junction [3]. In engineered multiphase materials, the organization and interaction of dissimilar constituents likewise contribute to mechanical behavior [1,2,4]. This is a functional and design analogy, not evidence that commercial RMCs reproduce the hierarchical architecture, adaptive behavior, or native biomechanics of teeth [5]. The conceptual connection to biomimetics is the relationship between constituent integration and function; the experimentally measured outcome here is composite repair bond strength. A complementary clinical motivation is to preserve a serviceable tooth–restoration complex through localized repair when appropriate [6,7]. Neither tissue preservation nor biological equivalence was measured in this experiment.

The tested materials represent distinct phase architectures. VITA Enamic is a polymer-infiltrated ceramic network (PICN) with an interconnected feldspathic ceramic skeleton, whereas GC Cerasmart and Shofu Block HC contain inorganic fillers dispersed in polymer matrices [8,9,10,11,12]. These manufacturer- and literature-described differences provide context for possible differences in conditioning response. They are not independently manipulated variables in the present design: material identity simultaneously incorporates differences in composition, filler characteristics, and processing. Previous conditioning studies report substrate-dependent bonding responses [13,14,15], supporting separate interpretation of each material.

Despite their favorable clinical properties, CAD/CAM RMC restorations may develop localized complications during service, including chipping, marginal fracture, cracking, or limited material loss [16]. Complete replacement of an otherwise serviceable restoration may require removal of additional restorative material and sound tooth structure and may increase treatment time, biological cost, and financial burden [6,7]. In contrast, intraoral composite repair confines treatment to the defective region and may prolong the functional service of the existing restoration [17,18,19,20]. Öztürk et al. [21] similarly discussed repair of CAD/CAM restorations within a tissue-preserving biomimetic framework. Importantly, the biomimetic relevance of repair does not imply regeneration of dental tissue or reproduction of a natural healing process. Rather, it lies in preserving as much of the existing tooth–restoration complex as possible through localized intervention. In this context, establishing an effective adhesive interface between the existing restoration and the repair composite is a prerequisite for conservative maintenance, although laboratory bond strength should not be interpreted as direct evidence of clinical tissue preservation or restoration of native tooth biomechanics.

Adhesion between an existing CAD/CAM substrate and a newly applied composite depends on the surface-conditioning and adhesive procedures [22]. The high degree of polymerization of prefabricated blocks limits the availability of unreacted groups for direct copolymerization, motivating mechanical and chemical pretreatment [23]. Investigated methods include HF etching, Al_2_O_3_ airborne-particle abrasion, tribochemical silica coating, and diamond bur roughening [24,25]. HF can dissolve susceptible glassy phases and modify the morphology available for resin infiltration and subsequent silane coupling [26]. Particle abrasion and bur roughening can create surface irregularities, but their bonding effects depend on the substrate and operating parameters [27]. Tribochemical treatment additionally offers a potential route for silica-mediated silane interaction. These are plausible treatment mechanisms; changes in roughness, surface energy, and chemistry require direct measurement and cannot be inferred quantitatively from SBS alone.

Available evidence remains heterogeneous. A scoping review identified substantial variation in experimental repair protocols [15], while individual studies evaluated different materials and conditioning approaches [28,29,30]. Andretti et al. [31] demonstrated material-dependent roughness responses to a multi-acid etchant using quantitative surface characterization, but did not test the repair SBS investigated here. Such findings motivate a material-specific assessment rather than an overall numerical ranking that pools structurally different substrates.

A further clinically relevant variable is the aging history of the restoration before repair. Unlike freshly manufactured laboratory specimens, restorations requiring intraoral repair have already been exposed to water, temperature fluctuations, functional loading, and other oral environmental challenges. In polymer-containing CAD/CAM materials, water sorption and hydrolytic processes may alter the polymer network and resin–filler interface, while differences in the coefficients of thermal expansion of constituent phases may generate repeated interfacial stresses during temperature changes [32,33,34]. Thermocycling is therefore widely used to reproduce selected aspects of hydrothermal aging under laboratory conditions. Importantly, pre-repair aging addresses a different scientific question from aging an already established repair interface. Whereas post-repair aging evaluates the stability of an existing bonded interface, pre-repair aging may alter the substrate itself before conditioning, potentially modifying its surface chemistry, micromechanical response, and susceptibility to subsequent treatment. The aging history of the restoration may therefore influence not only baseline substrate properties but also the effectiveness of the conditioning protocol subsequently applied.

Previous repair studies have considered thermal aging and conditioning, but differ in the materials, protocols, and aging sequences tested [22,29,35]. For example, Öztürk et al. [21] used a single airborne-particle abrasion protocol in additively and subtractively manufactured substrates. That design does not address the combined variation in substrate identity, alternative conditioning methods, and pre-repair aging investigated here. The remaining question is whether treatment-dependent differences in initial repair SBS vary with both material identity and prior substrate aging.

A full-factorial 3 × 5 × 2 design was therefore used to compare three RMCs, five surface-conditioning protocols, and two pre-repair aging conditions within one silane/adhesive/composite system. This design estimates main effects and interactions while permitting treatment comparisons within each material and aging condition. The biomimetic framework supplies a conceptual motivation for studying interfaces in materials with integrated, mechanically dissimilar constituents; it does not transform an SBS measurement into evidence of tissue preservation, regeneration, or recovery of native tooth biomechanics.

This in vitro study aimed to evaluate initial composite repair SBS to aged and non-aged VITA Enamic, GC Cerasmart, and Shofu Block HC substrates following five surface-conditioning protocols. Thermocycling comprised 25,000 cycles before repair; the repaired interfaces were not thermocycled or mechanically fatigued. The null hypotheses were that material type, surface-conditioning protocol, and pre-repair aging would not affect SBS, and that there would be no two- or three-way interactions among these factors.

## 2. Materials and Methods

### 2.1. Study Design and Sample Size

This in vitro study evaluated the bond strength of three resin-matrix computer-aided design/computer-aided manufacturing (CAD/CAM) materials with distinct microstructures to resin composite following different surface treatments, with and without pre-repair thermal aging. The materials were VITA Enamic (VITA Zahnfabrik, Bad Säckingen, Germany), a polymer-infiltrated ceramic network material; GC Cerasmart (GC Corporation, Tokyo, Japan), a resin nanoceramic; and Shofu Block HC (Shofu Dental Corporation, Kyoto, Japan), a zirconia–silica-containing material. Specimens were prepared at the Faculty of Dentistry, Muğla Sıtkı Koçman University. Thermal aging and shear bond strength (SBS) testing were performed in the research laboratories of the Faculty of Dentistry, Ankara University.

The sample size was determined using an a priori power analysis performed with G*Power 3.1.2 (Heinrich Heine University, Düsseldorf, Germany). The “ANOVA: fixed effects, special, main effects and interactions” option was selected for the material × surface treatment × thermal aging interaction. Assuming an effect size of f = 0.38, a significance level of α = 0.05, a statistical power of 1 − β = 0.95, 8 numerator degrees of freedom, and 30 experimental groups, the minimum required total sample size was calculated as 167. To maintain equal allocation across the 30 experimental cells, seven specimens were included in each subgroup, resulting in a total of 210 specimens for SBS testing.

This calculation targeted the specified omnibus interaction and does not guarantee adequate precision or power for every pairwise comparison.

A total of 210 specimens were prepared for SBS testing, comprising 70 specimens from each computer-aided design/computer-aided manufacturing (CAD/CAM) material. For each material, specimens were randomly allocated to thermally aged or non-aged groups and subsequently to five surface treatment groups (*n* = 7 per subgroup).

The surface treatment groups comprised a ground control, hydrofluoric acid (HF) etching, airborne-particle abrasion with aluminum oxide (Al_2_O_3_), tribochemical silica coating (CoJet), and mechanical roughening with a diamond bur. This design yielded 30 experimental subgroups (3 materials × 2 aging conditions × 5 surface treatments). Specimens were coded for shear bond strength (SBS) testing and failure mode assessment, and the evaluator was blinded to the material, surface treatment, and aging group. An additional specimen representing each of the 30 experimental conditions was prepared to evaluate surface treatment-induced micromorphological changes using scanning electron microscopy (SEM) (*n* = 30). Thus, 240 specimens were used in total: 210 for SBS testing and 30 for SEM analysis.

### 2.2. Materials Used

The compositions, lot numbers, and manufacturer details of the CAD/CAM materials and the materials used for surface treatment, silanization, adhesion, and repair are presented in Table 1. VITA Enamic is a polymer-infiltrated ceramic network (PICN) material comprising 86 wt% feldspathic ceramic and 14 wt% polymer based on urethane dimethacrylate (UDMA) and triethylene glycol dimethacrylate (TEGDMA). GC Cerasmart contains 71 wt% silica and barium glass nanofillers within a Bis-MEPP-, UDMA-, and DMA-based resin matrix. Shofu Block HC contains 61 wt% modified zirconium silicate nanofillers and silica within a UDMA/TEGDMA-based resin matrix.

### 2.3. Specimen Preparation

CAD/CAM blocks were sectioned into rectangular specimens measuring approximately 14 × 12 × 2 mm using a precision cutting machine (Secotom-60, Struers ApS, Ballerup, Denmark) under continuous water cooling. Specimen dimensions were verified using a digital caliper. Specimens that deviated from the target dimensions by more than ±0.05 mm, developed cracks during sectioning, or exhibited visible surface defects were excluded before testing and replaced as necessary. The documentation available for this revision does not specify the number or material distribution of specimens excluded during preparation; no retrospective estimates of these counts are provided. The final SBS sample comprised 210 specimens (*n* = 7 per experimental cell).

The specimens were embedded in 15 × 15 × 20 mm autopolymerizing acrylic resin blocks (İntegra, Birleşik Grup Dental, Ankara, Türkiye), leaving the test surfaces exposed. To standardize the surfaces before the experimental surface treatments, all specimens were ground with 1200-grit silicon carbide abrasive paper under water cooling at 150 rpm for 20 s.

### 2.4. Thermal Aging

Half of the SBS specimens (*n* = 105) underwent thermal aging before surface treatment, whereas the remaining half (*n* = 105) were non-aged. Thermal aging consisted of 25,000 cycles between distilled water baths at 5 °C and 55 °C, with a dwell time of 25 s in each bath and a transfer time of 10 s. The cycle count was selected from prior laboratory use [36] as an extended pre-repair thermal challenge. That reference evaluated lithium-disilicate/resin-cement bonding after storage and thermocycling; it does not validate an equivalent clinical service time for RMC substrates. The 5/55 °C bath range is used in dental laboratory aging protocols [21,36,37] to impose repeated thermal excursions and water exposure. Thermal expansion/contraction mismatch between constituent phases provides a rationale for this challenge; 55 °C was not selected for sterilization or as a polymer degradation threshold. Variability among laboratory regimens limits clinical extrapolation [32].

Thermocycling preceded surface conditioning and composite repair. The outcome was initial repair SBS after the 24 h water storage described below, not the durability of an already bonded repair interface.

### 2.5. Surface Treatment Protocols

Following the thermal aging phase, thermally aged and non-aged specimens of each material underwent one of five surface treatment protocols.

Ground control: No additional physical or chemical surface treatment was performed beyond the grinding and cleaning procedures used for standardization.

Hydrofluoric acid (HF): Specimen surfaces were treated with 9% HF (Ultradent Porcelain Etch, Ultradent Products Inc., South Jordan, UT, USA) for 60 s. The surfaces were then rinsed with a pressurized water spray for 1 min to remove residual acid completely.

Airborne-particle abrasion with aluminum oxide (Al_2_O_3_): Specimen surfaces were abraded with 50 µm Al_2_O_3_ particles using a laboratory sandblasting unit (Combilabor CL-FSG 3, Heraeus Kulzer, Germany) at 2.5 bar for 15 s. The nozzle was positioned perpendicular to the surface at a distance of 10 mm and moved in a circular motion. Following abrasion, the specimens were ultrasonically cleaned in deionized water for 15 min and air-dried.

Tribochemical silica coating: Specimen surfaces were treated with 30 µm silica-coated Al_2_O_3_ particles (CoJet Sand, 3M ESPE, St. Paul, MN, USA) at 2.5 bar for 15 s from a distance of approximately 10 mm. The specimens were then ultrasonically cleaned in deionized water for 15 min and air-dried.

Mechanical roughening with a diamond bur: Specimen surfaces were roughened with a green-banded diamond bur with a grit size of 125 µm (Hager & Meisinger GmbH, Neuss, Germany) at 200,000 rpm under water cooling for 10 s. The bur was applied in successive unidirectional strokes to cover the entire specimen surface and was replaced after every ten specimens. All procedures were performed by the same operator, who maintained similar manual pressure and movement patterns to reduce operator-related variability. The resulting surface debris was removed using an air–water spray for 20 s.

### 2.6. Silanization, Adhesive Application, and Composite Repair

Following surface treatment, the specimens were air-dried. A thin layer of Ultradent Silane (Ultradent Products Inc., South Jordan, UT, USA) was applied to all experimental surfaces, left for 60 s, and air-dried for 15 s. Scotchbond Universal Plus (3M Deutschland GmbH, Neuss, Germany) was then actively applied using a microapplicator for 20 s and air-thinned for 5 s. The adhesive layer was light-cured for 10 s in High Power mode using a Bluephase G2 light-emitting diode (LED) curing unit (Ivoclar Vivadent AG, Schaan, Liechtenstein) with a 10-mm-diameter light tip and a nominal irradiance of 1200 mW/cm^2^. During light curing, the tip was positioned perpendicular to and as close as possible to the specimen surface.

To standardize composite placement, cylindrical Teflon molds with an internal diameter of 5 mm and a height of 2 mm were positioned on the adhesive-treated surfaces. A nanohybrid resin composite (Filtek Z550, 3M ESPE, St. Paul, MN, USA) was placed in each mold as a single increment and light-cured for 20 s using the Bluephase G2 unit. After mold removal, the resin composite received an additional 20 s of light exposure from different directions. The specimens were stored in distilled water for 24 h before shear bond strength (SBS) testing.

### 2.7. Shear Bond Strength Testing

Shear bond strength (SBS) was measured using a universal testing machine (Lloyd Instruments, Fareham, Hampshire, England). Specimens were secured in the testing machine so that the load was applied parallel to and as close as possible to the interface between the restorative composite and the computer-aided design/computer-aided manufacturing (CAD/CAM) material. Loading was performed at a crosshead speed of 1 mm/min until bond failure occurred. The maximum force at failure was recorded in newtons (N). The bonded area was calculated as 19.63 mm^2^ using the formula A = πr^2^, based on the 5 mm diameter of the composite cylinder. SBS was calculated in megapascals (MPa) by dividing the maximum force at failure by the bonded area:

SBS (MPa) = P/A

where P is the maximum force at failure (N) and A is the bonded area (mm^2^).

### 2.8. Failure Mode Evaluation

After shear bond strength (SBS) testing, the fractured surfaces of all specimens were examined by the same evaluator using a Leica MZ12 stereomicroscope (Leica Microsystems, Heerbrugg, Switzerland) at ×20 magnification. Images were recorded using a Leica DFC450 digital camera. Failure modes were classified as adhesive when failure occurred at the interface between the resin composite and the CAD/CAM material, cohesive when failure occurred within either the resin composite or the CAD/CAM material, and mixed when both adhesive and cohesive failure were observed.

### 2.9. Scanning Electron Microscopy Analysis

A total of 30 specimens, prepared independently of the SBS specimens and representing one specimen per experimental condition, underwent scanning electron microscopy (SEM) analysis to assess micromorphological changes induced by surface treatment. Before examination, the specimens were coated with a gold–palladium alloy to prevent electrostatic charging and improve image quality. Images were acquired using a ZEISS EVO 40 scanning electron microscope (Carl Zeiss, Oberkochen, Germany) at ×2000 magnification. The SEM images were evaluated qualitatively for surface treatment–induced micromorphological changes and surface features. This qualitative protocol did not measure Ra, Sa, three-dimensional topography, contact angle, surface energy, or elemental composition.

### 2.10. Statistical Analysis

Statistical analyses were performed using IBM SPSS Statistics version 25.0 (IBM Corp., Armonk, NY, USA). Shear bond strength (SBS) data were expressed as means ± standard deviations. Analysis of variance (ANOVA) assumptions were assessed using model residuals. Residual normality was examined using Q–Q plots and the Shapiro–Wilk test, and homogeneity of variance was assessed using Levene’s test. Potentially influential observations were evaluated using standardized residuals and Cook’s distance. No assumption violations that would substantially compromise the validity of the analysis were identified.

The main effects of material type, surface treatment, and thermal aging on SBS, together with their two- and three-way interactions, were evaluated using three-way factorial ANOVA. Following the identification of a statistically significant three-way interaction, the simple effect of surface treatment was examined separately within each material × aging combination, with Bonferroni adjustment applied to pairwise comparisons. Thermally aged and non-aged conditions were also compared within each material × surface treatment combination using Bonferroni-adjusted pairwise comparisons.

Additional descriptive estimates were calculated from the rounded group means, SDs, and *n* = 7 in Table 2 for all 15 aging contrasts (non-aged minus thermally aged) and six HF–CoJet contrasts. For independent groups, the unadjusted 95% CI for the mean difference Δ was Δ ± t(0.975, ν) × √(s_1_^2^/n_1_ + s_2_^2^/n_2_), with Welch–Satterthwaite degrees of freedom ν = (s_1_^2^/n_1_ + s_2_^2^/n_2_)^2^/[(s_1_^2^/n_1_)^2^/(n_1_ − 1) + (s_2_^2^/n_2_)^2^/(n_2_ − 1)]. Hedges’ g was calculated as J × Δ/s_p_, with pooled SD s_p_ = √[((n_1_ − 1)s_1_^2^ + (n_2_ − 1)s_2_^2^)/(n_1_ + n_2_ − 2)] and exact small-sample factor J = Γ(ν_p_/2)/[√(ν_p_/2)Γ((ν_p_ − 1)/2)], where ν_p_ = n_1_ + n_2_ − 2 = 12. These summary-based estimates are approximate because the inputs are rounded; the intervals describe uncertainty in the mean difference, not in g. They are neither multiplicity-adjusted nor model-based intervals for the original Bonferroni tests. No original *p* value was replaced, and nonsignificance was not interpreted as equivalence. Raw observations would be required to repeat residual, influence, bootstrap, or robust-model analyses.

Failure modes were classified as adhesive, cohesive, or mixed. Within each material, the association between thermal aging status and failure mode distribution was evaluated using the chi-square test. No inferential comparisons of failure mode distributions were performed among surface treatment groups. The statistical significance level was set at α = 0.05.

## 3. Results

### 3.1. Shear Bond Strength

**Figure 1:**
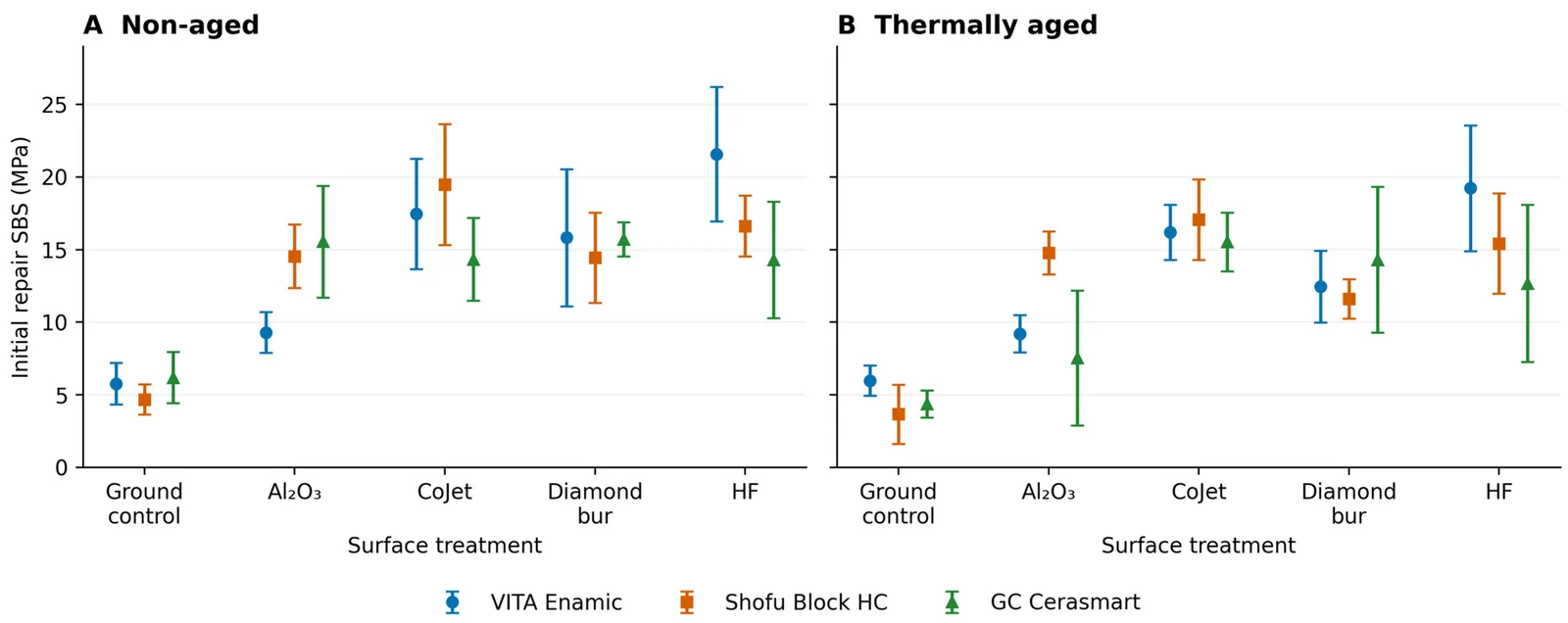
Initial repair shear bond strength (SBS) by categorical surface treatment and material. (**A**) Non-aged substrates; (**B**) thermally aged substrates (25,000 cycles before repair). Unconnected symbols and error bars represent means ± SD (*n* = 7 per cell); horizontal offsets distinguish materials and have no numerical meaning. HF, hydrofluoric acid; Al_2_O_3_, aluminum oxide abrasion; CoJet, tribochemical silica coating. Error bars are descriptive and do not encode pairwise statistical significance.

Shear bond strength (SBS) values varied according to material type, surface treatment, and thermal aging status. The means and standard deviations for all experimental groups are presented in Table 2. Mean SBS values ranged from 3.66 to 19.23 MPa in the thermally aged groups and from 4.68 to 21.58 MPa in the non-aged groups. Under both aging conditions, the lowest mean SBS values were observed in the ground control groups that received no additional surface treatment (Figure 1).

Three-way factorial analysis of variance (ANOVA) revealed statistically significant main effects of material type (F = 3.666; *p* = 0.027), surface treatment (F = 101.826; *p* < 0.001), and thermal aging (F = 16.476; *p* < 0.001) on SBS. Surface treatment had the largest effect size (partial η^2^ = 0.694). The material × surface treatment interaction was also statistically significant (F = 8.043; *p* < 0.001; partial η^2^ = 0.263). Neither the material × thermal aging interaction (*p* = 0.592) nor the surface treatment × thermal aging interaction (*p* = 0.506) was statistically significant. However, the material × surface treatment × thermal aging interaction was statistically significant (F = 2.427; *p* = 0.016; partial η^2^ = 0.097) (Table 3).

Bonferroni-adjusted aging comparisons identified a statistically significant reduction only for GC Cerasmart treated with Al_2_O_3_: 15.55 ± 3.85 MPa in non-aged specimens versus 7.54 ± 4.64 MPa in thermally aged specimens (non-aged minus thermally aged mean difference, 8.01 MPa; adjusted *p* < 0.001). No statistically significant aging difference was detected in the remaining 14 material × surface-treatment combinations (all adjusted *p* > 0.05). These pairwise findings describe the observed pattern accompanying the significant three-way interaction; they are not a formal decomposition of its source.

For thermally aged VITA Enamic specimens, HF yielded significantly higher SBS than Al_2_O_3_ abrasion, diamond bur roughening, and the ground control, whereas CoJet yielded significantly higher SBS than Al_2_O_3_ abrasion and the ground control. Diamond bur roughening also yielded significantly higher SBS than the ground control. No statistically significant difference was detected between HF and CoJet. These findings were consistent with the pairwise comparisons summarized in Table 4. For non-aged VITA Enamic specimens, hydrofluoric acid (HF) etching, tribochemical silica coating (CoJet), and mechanical roughening with a diamond bur yielded statistically significantly higher SBS values than airborne-particle abrasion with Al_2_O_3_ and the ground control condition. No statistically significant difference was observed between the HF and CoJet groups (*p* = 0.374), whereas the HF group exhibited statistically significantly higher SBS than the diamond bur group (*p* = 0.047).

For Shofu Block HC, all active treatments yielded significantly higher SBS than the ground control in thermally aged specimens (*p* < 0.001), and CoJet yielded higher SBS than diamond bur roughening (*p* = 0.002). No statistically significant differences were detected in the remaining comparisons among active treatments. In non-aged specimens, all active treatments also yielded higher SBS than the ground control; CoJet yielded higher SBS than Al_2_O_3_ (*p* = 0.020) and diamond bur roughening (*p* = 0.017). No statistically significant difference was detected between CoJet and HF (*p* = 0.598).

For GC Cerasmart, CoJet and diamond bur roughening yielded significantly higher SBS than Al_2_O_3_ in thermally aged specimens (*p* = 0.008 and *p* = 0.038, respectively). No statistically significant differences were detected among CoJet, diamond bur, and HF. The ground control yielded lower SBS than CoJet, diamond bur, and HF; its difference from Al_2_O_3_ was not statistically significant. In non-aged specimens, all active treatments yielded higher SBS than the ground control (*p* < 0.001), with no statistically significant differences among the four active treatments.

Summary-based estimates are reported in Table 5 and Table 6. For Al_2_O_3_-treated GC Cerasmart, the non-aged minus thermally aged difference was 8.01 MPa (descriptive unadjusted 95% CI, 3.03–12.99 MPa; Hedges’ g = 1.76). The HF–CoJet intervals included zero in all six material × aging combinations. Some unadjusted aging intervals excluded zero despite nonsignificant original Bonferroni comparisons; these estimates do not change the reported adjusted significance decisions.

### 3.2. Failure Modes

**Figure 2:**
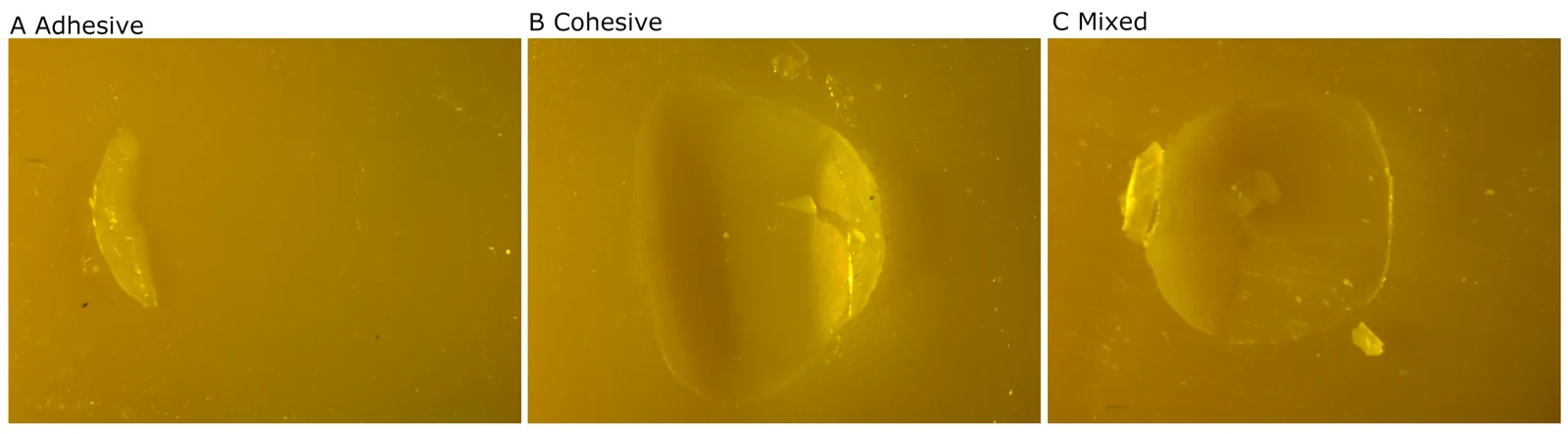
Representative failure modes observed after shear bond strength testing: (**A**) adhesive failure (Type 1); (**B**) cohesive failure (Type 2); and (**C**) mixed failure (Type 3).

No statistically significant difference in failure mode distribution was detected between pre-repair aging conditions for VITA Enamic (χ^2^(2) = 2.976; *p* = 0.226), GC Cerasmart (χ^2^(2) = 4.500; *p* = 0.105), or Shofu Block HC (χ^2^(2) = 0.517; *p* = 0.772). Because no inferential comparisons were performed among surface treatment groups, no conclusions were drawn regarding the effect of surface treatment on failure mode distribution. Representative failure modes are shown in Figure 2; pooled frequencies and the original aging comparisons are reported in Table 7.

### 3.3. Scanning Electron Microscopy Findings

**Figure 3:**
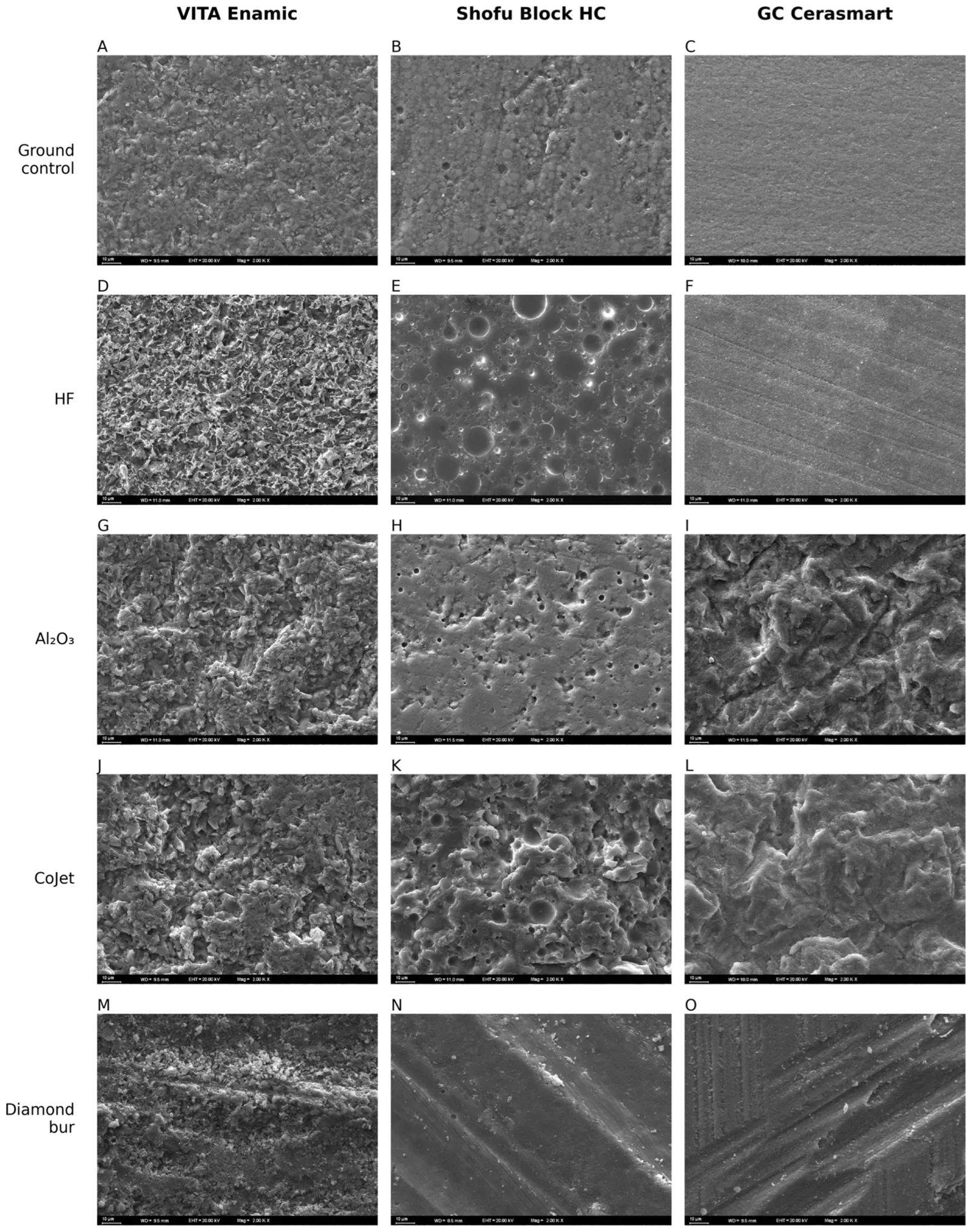
Representative non-aged surfaces after conditioning (SEM, original acquisition ×2000; original 10 µm scale bars retained). Columns: VITA Enamic, Shofu Block HC, and GC Cerasmart. Rows: ground control (**A**–**C**), HF (**D**–**F**), Al_2_O_3_ (**G**–**I**), CoJet (**J**–**L**), and diamond bur (**M**–**O**). One specimen per condition; images illustrate qualitative morphology and do not provide quantitative roughness or chemical measurements.

**Figure 4:**
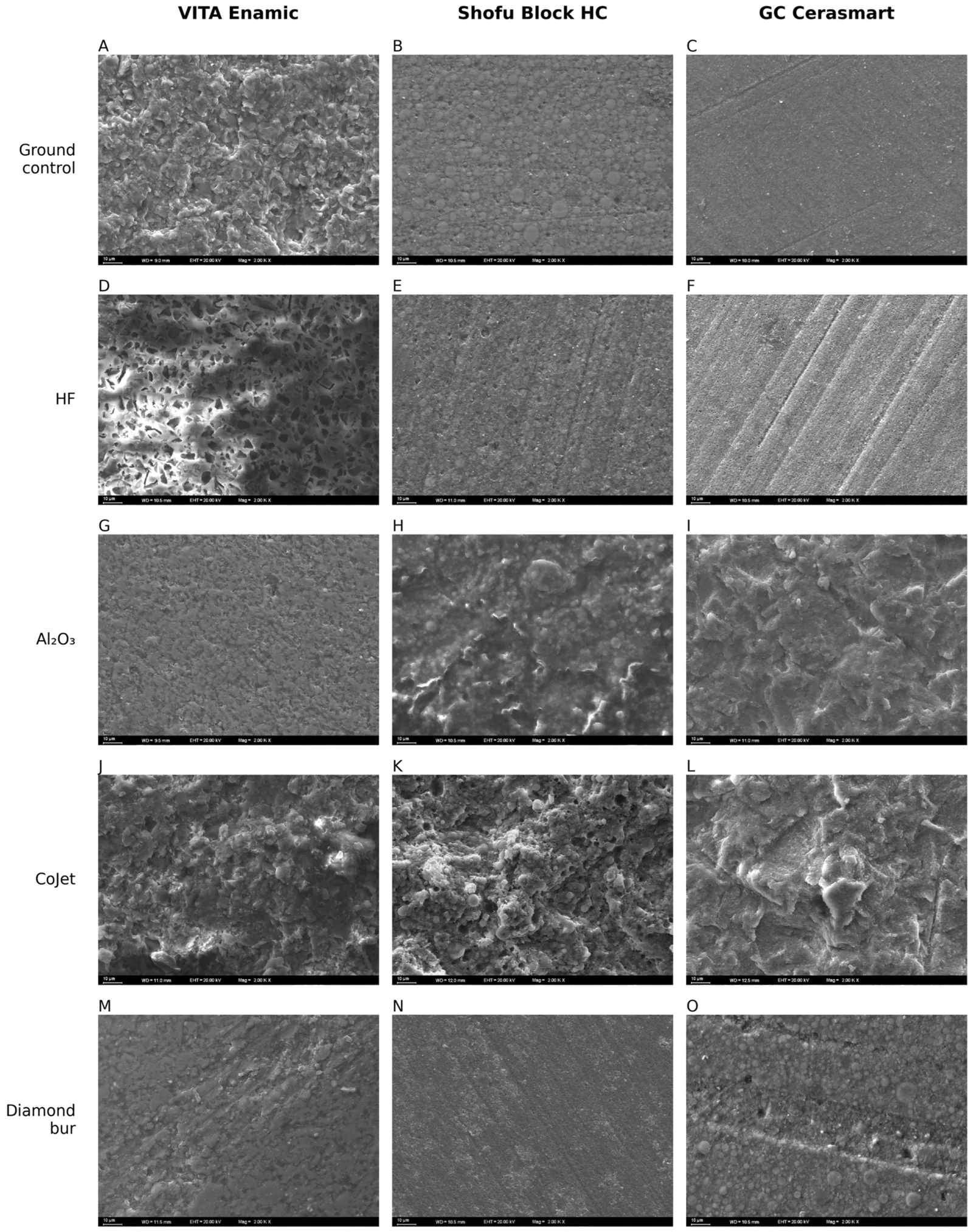
Representative thermally aged surfaces after conditioning (SEM, original acquisition ×2000; original 10 µm scale bars retained). Substrates received 25,000 thermal cycles before conditioning. Material columns and treatment rows follow Figure 3: ground control (**A**–**C**), HF (**D**–**F**), Al_2_O_3_ (**G**–**I**), CoJet (**J**–**L**), and diamond bur (**M**–**O**). One specimen per condition; no quantitative or inferential image comparisons were performed.

The representative SEM images showed porous network-like features on HF-treated VITA Enamic, particularly in non-aged specimens, and crater-like depressions on non-aged HF-treated Shofu Block HC. Al_2_O_3_-treated surfaces displayed irregular features, CoJet-treated surfaces included particle-like features, and diamond bur-treated surfaces exhibited directional grooves. These descriptions concern visible morphology only. Particle composition, filler exposure or loss, and the magnitude of roughness or micromechanical retention cannot be established from these images. One independent specimen was examined per condition; no quantitative or inferential SEM comparisons were performed (Figure 3 and Figure 4).

## 4. Discussion

### 4.1. Principal Findings and Statistical Precision

Surface treatment had the largest main effect on initial repair SBS (F = 101.826, *p* < 0.001, partial η^2^ = 0.694), compared with pre-repair aging (partial η^2^ = 0.084) and material identity (partial η^2^ = 0.039). The material × surface treatment interaction (partial η^2^ = 0.263) indicates that treatment contrasts should be interpreted within materials. The smaller three-way interaction (F = 2.427, *p* = 0.016, partial η^2^ = 0.097) further qualifies these comparisons. Partial η^2^ describes statistical effect magnitude conditional on this model; it does not identify a physical adhesion mechanism or partition the contribution of roughness and chemistry.

The null hypotheses of no material, surface-treatment, or pre-repair aging main effects were rejected. The hypothesis of no interactions was also rejected because the material × surface treatment and three-way interactions were significant. No statistically significant material × aging or surface treatment × aging interaction was detected. These latter results do not establish absence of interaction.

Material-dependent conditioning has also been examined in repair syntheses [38] and dentin-bonding experiments [39], while a network meta-analysis compared ceramic pretreatments [40]. These studies address different substrate/interface combinations and cannot establish equivalence to the present repair protocol. Their broader findings support attention to material and protocol differences, while the present experiment supplies within-material comparisons under one controlled repair system.

The post-hoc pattern was particularly associated with Al_2_O_3_-treated GC Cerasmart: the non-aged minus thermally aged difference was 8.01 MPa (descriptive, unadjusted 95% CI, 3.03–12.99 MPa; Hedges’ g = 1.76). It was the only aging contrast significant in the reported Bonferroni-adjusted comparisons. This does not demonstrate that all other aging effects are zero, nor does it formally establish that this cell alone generated the omnibus interaction. We therefore interpret the interaction as a localized pattern requiring replication, rather than evidence of strong aging dependence across all materials.

The a priori calculation addressed an assumed omnibus interaction effect, not the precision or power of every pairwise comparison. With *n* = 7 per cell and variable SDs, several differences remain imprecisely estimated (Table 5 and Table 6). The unadjusted Welch intervals use the variances of the two compared groups, whereas the original factorial comparisons use their stated model and multiplicity procedure. An interval excluding zero can therefore coexist with a nonsignificant adjusted comparison; it must not be used to override that comparison. Bonferroni adjustment reduces false-positive risk across comparisons but also reduces sensitivity. These summary-based estimates supplement the original analysis; they do not constitute a raw-data robustness, outlier, or sensitivity reanalysis.

### 4.2. VITA Enamic

For VITA Enamic, HF yielded higher SBS than Al_2_O_3_, diamond bur, and ground control under both aging conditions. CoJet yielded higher SBS than Al_2_O_3_ and control, but no statistically significant HF–CoJet difference was detected. HF had the numerically highest mean SBS under both aging conditions, but this descriptive ranking does not establish superiority over CoJet. The HF-minus-CoJet descriptive intervals were −1.05 to 7.13 MPa in thermally aged specimens and −0.85 to 9.07 MPa in non-aged specimens (Table 6), allowing effects of potentially relevant magnitude in either direction.

The PICN architecture supplies a plausible context: HF can attack a susceptible glassy phase, and subsequent silanization can couple silica-containing surfaces to resin [1,2,26]. The network-like SEM appearance is consistent with this mechanism but does not quantify dissolution, infiltration, or silane bonding. Lechte et al. [41] also evaluated pretreatment and primer use for aged PICN, supporting consideration of both substrate and adhesive strategy. Differences in primer and test protocols prevent direct equivalence. Although CoJet offers another conditioning route, neither silica enrichment nor superiority of one chemical pathway was measured here.

### 4.3. Shofu Block HC

For Shofu Block HC, CoJet exceeded diamond bur roughening after pre-repair aging and exceeded both diamond bur and Al_2_O_3_ in non-aged specimens. All active treatments exceeded control under both conditions. CoJet had the numerically highest mean SBS under both aging conditions; however, no statistically significant difference was detected between CoJet and HF. The HF-minus-CoJet intervals were −5.32 to 2.02 MPa in thermally aged specimens and −6.85 to 1.15 MPa in non-aged specimens. These estimates support cautious interpretation and do not establish equivalence or identify a uniquely superior treatment.

Its filler-containing polymer matrix differs from the continuous ceramic network of VITA Enamic [8,9,10,11,12]. Abrasive impact and possible silane interaction after tribochemical treatment may contribute to bonding, while HF response depends on the accessibility and susceptibility of inorganic phases. Prior repair studies investigated conditioning and universal adhesive combinations in hybrid CAD/CAM materials [22,23,24,25]; their protocols differ from the fixed adhesive system used here. The present experiment cannot separate filler type, amount, or distribution from other product differences. The particle-like SEM appearance does not demonstrate the amount, composition, or persistence of deposited silica.

### 4.4. GC Cerasmart

For GC Cerasmart, no statistically significant differences among active treatments were detected in non-aged specimens. After pre-repair aging, CoJet and diamond bur roughening exceeded Al_2_O_3_, while the Al_2_O_3_–control contrast was nonsignificant. This is the specific treatment pattern accompanying the 8.01 MPa aging-associated decrease in the Al_2_O_3_ group; it should not be generalized to all GC Cerasmart treatments or to the other materials.

Water exposure and thermal cycling could alter a polymer-containing substrate or its filler–matrix interfaces before subsequent abrasion [33,34]. An altered response to particle impact is consequently plausible, but subsurface damage, polymer plasticization, and interfacial chemistry were not measured. Because abrasion occurred after aging, the experiment does not demonstrate water penetration into abrasion-created defects during thermocycling. Suta et al. [30] found that sandblasting combined with a 10-MDP/silane-containing adhesive favored Cerasmart repair in a different protocol. This does not contradict the present aging-dependent Al_2_O_3_ finding: the adhesive strategy, aging sequence, and microtensile outcome differ. Likewise, the nonsignificant GC Cerasmart–CoJet aging contrast does not prove preserved bonding or resistance to aging.

Diamond bur roughening provides a mechanically different route through directional grooves rather than particle impact. Its higher SBS than Al_2_O_3_ in aged GC Cerasmart is a supported contrast, whereas its numerical lead in non-aged GC Cerasmart is not a demonstrated advantage over other active treatments. Arkoy and Ulusoy [23] examined alternative roughening procedures, emphasizing that outcomes depend on the entire conditioning protocol. Bur-generated features may contribute to retention, but manual pressure, grit, speed, and duration limit extrapolation beyond the tested procedure.

### 4.5. Thermal Challenge and Interpretation Boundaries

The 25,000-cycle regimen was an extended laboratory pre-repair challenge. Pilecco et al. [36] used that cycle count with lithium disilicate, resin cement, and additional water storage after bonding; this is laboratory precedent, not validation of the present substrate-aging model or a conversion to clinical years. Dental protocols differ in bath temperatures, dwell times, transfer intervals, geometry, and aging sequence [32]. The 5/55 °C range supplies repeated thermal excursions, as used in other dental laboratory studies [21,36,37]; sterilization and attainment of a polymer degradation threshold were not its purposes. Because shorter and intermediate cycle counts were not tested, sensitivity to aging intensity remains unknown.

All thermocycling occurred before conditioning and repair. SBS was assessed after 24 h water storage, so the findings address initial bonding to previously aged substrates. Öztürk et al. [21] included both pre-repair and post-repair thermocycling in a different material/repair system; that design illustrates why aging sequence must be specified. The present experiment provides no evidence on post-repair thermal stability, mechanical fatigue resistance, restoration survival, or long-term clinical superiority.

Failure-mode comparisons were retained within their original scope: aging groups pooled across treatments within each material. Surface-treatment-specific failure frequencies were not tested inferentially. Cohesive or mixed failure cannot establish a causal explanation for treatment differences because the substrate, loading geometry, and stress distribution also influence fracture. Moreover, the VITA Enamic chi-square comparison contains sparse expected counts, limiting the reliability of its asymptotic *p* value; it should not be treated as evidence of equivalent failure distributions.

SEM contributes qualitative morphological context, not quantitative proof of roughness-controlled adhesion. Andretti et al. [31] used 3D profilometry alongside SEM and found material-dependent responses to a different etchant. Such complementary measurements are needed to test a topography-based explanation. The present two-dimensional images from one specimen per condition cannot yield validated retrospective Ra or Sa values, quantify silica deposition, or establish surface-energy differences. Chemical and topographic contributions also cannot be ranked because all groups received the same silane and adhesive and these contributions were not independently isolated.

### 4.6. Conceptual Relevance and Limitations

The biomimetic connection remains a conceptual framework: mechanically dissimilar but integrated constituents provide a biological design analogy, and localized repair is motivated by preserving a serviceable tooth–restoration complex [3,5,6,7,17,18,19,20]. Compositional heterogeneity alone is not proof of biomimicry. The experiment measured SBS between artificial substrates and repair composite; it did not measure tissue preservation, regeneration, natural healing, or restoration of native tooth biomechanics. Its practical contribution is a bounded comparison of conditioning responses under one laboratory protocol.

Limitations include the in vitro design; *n* = 7 per cell with high variability in some groups; numerous pairwise comparisons and limited precision despite multiplicity adjustment; and aging only before repair, without post-repair thermal or mechanical fatigue. SBS loading creates nonuniform interfacial stresses and may involve cohesive failure, so the measured stress is not a pure interfacial material property. Only one silane, adhesive, and repair composite system was used. SEM included one specimen per condition and was qualitative, without quantitative roughness, contact angle, surface energy, or elemental characterization. Product composition was not independently measured or experimentally separated from material identity. The 25,000-cycle regimen has no validated clinical-time equivalence for these materials, and a numerical breakdown of exclusions during specimen preparation is unavailable in the documentation reviewed for this revision. These boundaries limit mechanistic and clinical extrapolation without invalidating the comparisons actually performed.

Future studies should replicate the material-specific contrasts with sample sizes chosen for pairwise precision, examine several pre-repair aging intensities, and test post-repair thermocycling and mechanical fatigue. Replicated profilometry, AFM or validated 3D surface measurements, contact-angle measurements, and elemental analysis should be combined with different adhesive systems to distinguish plausible mechanisms from measured pathways.

## 5. Conclusions

Surface treatment was the largest main effect on initial repair SBS (partial η^2^ = 0.694). Significant material × surface treatment (*p* < 0.001) and three-way (*p* = 0.016) interactions were accompanied by distinct pairwise patterns: under both aging conditions, HF yielded significantly higher SBS than Al_2_O_3_ and diamond bur for VITA Enamic, whereas CoJet yielded significantly higher SBS than diamond bur for Shofu Block HC; HF–CoJet differences were not statistically significant in either material. For GC Cerasmart, CoJet and diamond bur exceeded Al_2_O_3_ only in thermally aged substrates, and the only statistically significant aging contrast was the Al_2_O_3_-associated decrease from 15.55 to 7.54 MPa (adjusted *p* < 0.001). No single universally optimal surface-conditioning protocol was identified across the tested materials under these experimental conditions. These findings concern initial repair bonding after pre-repair substrate aging and do not establish post-repair durability or clinical superiority.

## Figures and Tables

**Table 1:** Main characteristics of the CAD/CAM materials and the materials used for surface treatment, silanization, adhesion, and repair.

Material	Main Composition	Lot Number	Manufacturer
VITA Enamic	86% feldspathic ceramic; 14% UDMA/TEGDMA	221630	VITA Zahnfabrik, Bad Säckingen, Germany
GC Cerasmart	Bis-MEPP, UDMA, DMA; 71% silica and barium glass nanofillers	2404221	GC Corporation, Tokyo, Japan
Shofu Block HC	61% modified zirconium silicate nanofillers and silica; UDMA/TEGDMA	0323758	Shofu Dental Corporation, Kyoto, Japan
Korox	99.7% Al_2_O_3_; 50 µm	201408	BEGO, Bremen, Germany
Ultradent Porcelain Etch	9% buffered HF	4518	Ultradent Products Inc., South Jordan, UT, USA
CoJet Sand	Silica-modified Al_2_O_3_ particles, 30 µm	654604	3M ESPE, St. Paul, MN, USA
Ultradent Silane	γ-MPS, isopropyl alcohol	626112	Ultradent Products Inc., South Jordan, UT, USA
Diamond bur	Diamond particles, 125 µm	250308	Hager & Meisinger GmbH, Neuss, Germany
Scotchbond Universal Plus	10-MDP, HEMA, Vitrebond copolymer, silane, silica filler, ethanol, and water	11805468	3M Deutschland GmbH, Neuss, Germany
Filtek Z550	Bis-GMA, UDMA, Bis-EMA, PEGDMA, TEGDMA, and nanofillers	10887780	3M ESPE, St. Paul, MN, USA

Abbreviations: UDMA, urethane dimethacrylate; TEGDMA, triethylene glycol dimethacrylate; Bis-MEPP, 2,2-bis(4-methacryloxypolyethoxyphenyl) propane; DMA, dimethacrylate; Al_2_O_3_, aluminum oxide; HF, hydrofluoric acid; γ-MPS, γ-methacryloxypropyltrimethoxysilane; 10-MDP, 10-methacryloyloxydecyl dihydrogen phosphate; HEMA, 2-hydroxyethyl methacrylate; Bis-GMA, bisphenol A glycidyl dimethacrylate; Bis-EMA, ethoxylated bisphenol A dimethacrylate; PEGDMA, polyethylene glycol dimethacrylate.

**Table 2:** Shear bond strength (SBS) values according to material, surface treatment, and pre-repair thermal aging status.

Material	Surface Treatment	Thermally Aged Mean ± SD	Non-Aged Mean ± SD
GC Cerasmart	Ground Control	4.36 ± 0.93	6.19 ± 1.76
	Al_2_O_3_	7.54 ± 4.64	15.55 ± 3.85
	Cojet	15.53 ± 2.01	14.33 ± 2.85
	Diamond Bur	14.30 ± 5.02	15.71 ± 1.18
	HF	12.68 ± 5.41	14.30 ± 4.02
Shofu Block HC	Ground Control	3.66 ± 2.03	4.68 ± 1.05
	Al_2_O_3_	14.77 ± 1.48	14.55 ± 2.20
	Cojet	17.07 ± 2.77	19.48 ± 4.17
	Diamond Bur	11.60 ± 1.36	14.44 ± 3.10
	HF	15.42 ± 3.46	16.63 ± 2.10
VITA Enamic	Ground Control	5.98 ± 1.05	5.77 ± 1.43
	Al_2_O_3_	9.21 ± 1.27	9.30 ± 1.39
	Cojet	16.19 ± 1.90	17.47 ± 3.80
	Diamond Bur	12.45 ± 2.48	15.82 ± 4.72
	HF	19.23 ± 4.32	21.58 ± 4.64

Data are mean ± standard deviation (SD), in MPa; *n* = 7 per cell. Thermally aged indicates 25,000 cycles before repair; non-aged indicates no thermocycling. HF, hydrofluoric acid; Al_2_O_3_, aluminum oxide abrasion; CoJet, tribochemical silica coating.

**Table 3:** Three-way factorial analysis of variance (ANOVA) results for the main effects and interactions of material type, surface treatment, and thermal aging on shear bond strength (SBS).

Effect	df	F	*p*	Partial η^2^
Material	2	3.666	0.027	0.039
Surface treatment	4	101.826	<0.001	0.694
Thermal aging	1	16.476	<0.001	0.084
Material × surface treatment	8	8.043	<0.001	0.263
Material × thermal aging	2	0.526	0.592	0.006
Surface treatment × thermal aging	4	0.833	0.506	0.018
Material × surface treatment × thermal aging	8	2.427	0.016	0.097

df, degrees of freedom; F, F statistic; partial η^2^, partial eta squared. Error degrees of freedom = 180. Statistical significance was set at *p* < 0.05.

**Table 4:** Summary of statistically significant Bonferroni-adjusted pairwise comparisons of surface treatments according to material and thermal aging status.

Material	Condition	Statistically Significant Comparisons
VITA Enamic	Thermally aged	HF > Al_2_O_3_, diamond bur, ground control; CoJet > Al_2_O_3_, ground control; diamond bur > ground control
	Non-aged	HF > Al_2_O_3_, diamond bur, ground control; CoJet > Al_2_O_3_, ground control; diamond bur > Al_2_O_3_, ground control
Shofu Block HC	Thermally aged	All active surface treatments > ground control; CoJet > diamond bur
	Non-aged	All active surface treatments > ground control; CoJet > Al_2_O_3_, diamond bur
GC Cerasmart	Thermally aged	CoJet > Al_2_O_3_; diamond bur > Al_2_O_3_; CoJet, diamond bur, and HF > ground control
	Non-aged	Al_2_O_3_, CoJet, diamond bur, and HF > ground control; no statistically significant differences among active surface treatments

The “>” symbol identifies a statistically significant Bonferroni-adjusted difference (*p* < 0.05). The final row also records the absence of statistically significant differences among the active treatments. Thermally aged and non-aged refer to substrate status before repair. HF, hydrofluoric acid; Al_2_O_3_, aluminum oxide abrasion; CoJet, tribochemical silica coating.

**Table 5:** Descriptive aging contrasts from rounded summary statistics.

Material	Surface Treatment	Difference Mpa	Unadjusted 95% CI Mpa	Hedges’ G
GC Cerasmart	Ground control	1.83	0.13 to 3.53	1.22
	Al_2_O_3_	8.01	3.03 to 12.99	1.76
	CoJet	−1.20	−4.11 to 1.71	−0.46
	Diamond bur	1.41	−3.25 to 6.07	0.36
	HF	1.62	−3.98 to 7.22	0.32
Shofu Block HC	Ground control	1.02	−0.93 to 2.97	0.59
	Al_2_O_3_	−0.22	−2.44 to 2.00	−0.11
	CoJet	2.41	−1.78 to 6.60	0.64
	Diamond bur	2.84	−0.10 to 5.78	1.11
	HF	1.21	−2.20 to 4.62	0.40
VITA Enamic	Ground control	−0.21	−1.69 to 1.27	−0.16
	Al_2_O_3_	0.09	−1.46 to 1.64	0.06
	CoJet	1.28	−2.36 to 4.92	0.40
	Diamond bur	3.37	−1.18 to 7.92	0.84
	HF	2.35	−2.87 to 7.57	0.49

Difference = non-aged minus thermally aged. *n* = 7 per group. CIs are unadjusted Welch intervals for mean differences; Hedges’ g uses a pooled SD and exact small-sample correction. Estimates are approximate because source means and SDs are rounded. These descriptive intervals do not replace the original Bonferroni-adjusted tests.

**Table 6:** Descriptive HF-minus-CoJet contrasts from rounded summary statistics.

Material	Substrate Status	Difference Mpa	Unadjusted 95% CI Mpa	Hedges’ G
GC Cerasmart	Thermally aged	−2.85	−7.92 to 2.22	−0.65
	Non-aged	−0.03	−4.14 to 4.08	−0.01
Shofu Block HC	Thermally aged	−1.65	−5.32 to 2.02	−0.49
	Non-aged	−2.85	−6.85 to 1.15	−0.81
VITA Enamic	Thermally aged	3.04	−1.05 to 7.13	0.85
	Non-aged	4.11	−0.85 to 9.07	0.91

Difference = HF minus CoJet within each material and aging condition. *n* = 7 per group. CIs are unadjusted Welch intervals for mean differences; Hedges’ g uses a pooled SD and exact small-sample correction. Estimates are approximate because source means and SDs are rounded. These descriptive intervals do not replace the original Bonferroni-adjusted tests.

**Table 7:** Failure mode distributions according to material and pre-repair thermal aging status.

Material	Aging Status	Adhesive, n (%)	Cohesive, n (%)	Mixed, n (%)	χ^2^	*p*
VITA Enamic	Non-aged	3 (8.6)	18 (51.4)	14 (40.0)	2.976	0.226
	Thermally aged	2 (5.7)	25 (71.4)	8 (22.9)	—	—
GC Cerasmart	Non-aged	3 (8.6)	17 (48.6)	15 (42.9)	4.500	0.105
	Thermally aged	9 (25.7)	17 (48.6)	9 (25.7)	—	—
Shofu Block HC	Non-aged	6 (17.1)	15 (42.9)	14 (40.0)	0.517	0.772
	Thermally aged	5 (14.3)	18 (51.4)	12 (34.3)	—	—

Data are frequencies and row percentages. Each material–aging group comprised 35 specimens pooled across five surface treatments. Pearson’s chi-square statistics compare the two aging groups within each material (df = 2); each statistic and *p* value is shown once. No inferential comparisons among surface treatments were performed. For VITA Enamic, two of six expected counts were 2.5, so its asymptotic *p* value should be interpreted cautiously. The original test statistics and *p* values are retained.

## Data Availability

The raw experimental data and the complete set of original, uncropped scanning electron microscopy (SEM) images supporting the findings of this study are available from the corresponding author upon reasonable request.
